# Scalable optical metasurfaces for ultrasensitive, label-free and real-time biosensing

**DOI:** 10.1007/s44258-026-00090-w

**Published:** 2026-07-14

**Authors:** Hao Wang, Nanzhong Deng, Yue Xiao, Ashish Pandey, Shunzhi Wang, Haogang Cai

**Affiliations:** 1 Tech4Health Institute, New York University Grossman School of Medicine, Queens, NY USA; 2 Department of Radiology, New York University Grossman School of Medicine, New York, NY USA; 3 Department of Biomedical Engineering, New York University, Brooklyn, NY USA; 4 Institute for Systems Genetics, New York University Grossman School of Medicine, New York, NY USA

**Keywords:** Optical metasurfaces, Label-free biosensing, Optofluidic integration, Scalable nanopatterning, Nanosphere lithography, Nanophotonics

## Abstract

Novel biosensors based on optical metasurfaces offer superior sensing performance in compact form factors, with transformative potential spanning biomedical research, clinical diagnostics and drug screening. However, their translation to real-world applications is hindered by a strong reliance on sophisticated and expensive top-down nanolithographic techniques with limited accessibility and low throughput. Here, we demonstrate scalable, high-throughput metasurfaces with optofluidic integration for label-free biosensing, exemplified by high-quality large-area gold nanohole arrays via nanosphere self-assembly lithography. Through comprehensive resonance analysis, we identified a surface plasmon polariton–Bloch wave mode that is both highly sensitive and experimentally accessible using cost-effective setups with incoherent visible light. Moreover, to improve the mass transport and analyte capture, we optimized the functionalization scheme, microfluidic design, and the metasurface placement within the microfluidic channel. The versatile meta-sensors were demonstrated with a broad range of targets, from biomolecules such as immunoglobulin G, streptavidin, to streptavidin-coated nanoparticles, which mimic virus particles and extracellular vesicles. By holistically improving the nanopatterning quality, functionalization efficiency and optofluidic integration, we achieved an experimental refractometric sensitivity of 498 nm/refractive index unit at 736 nm wavelength, a limit of detection of 0.17 ng/mL for biomolecules and ≲1 × 10^7^ /mL for nanoparticles. These scalable, cost-effective meta-sensors deliver sensing performance, dynamic range, and stability comparable to, or even surpass, those of state-of-the-art devices fabricated using top-down lithography, thereby bridging the gap toward practical applications.

**Graphical Abstract:**

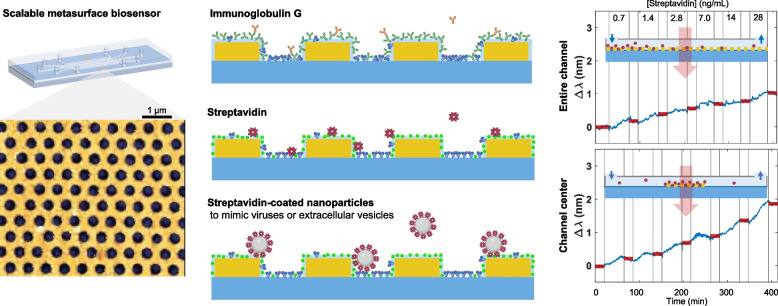

**Supplementary Information:**

The online version contains supplementary material available at 10.1007/s44258-026-00090-w.

## Highlights

- Scalable, versatile optical sensors detect a broad range of biomolecules and particles without labels in real time.
- Integrated microfluidics boosts delivery and capture of rare targets on light-enhanced sensor surfaces.
- Sensors exceed leading devices while using low-cost, high-throughput manufacturing process and visible light setups.
- Cleanroom-free method makes advanced biosensing more accessible and affordable for real-world applications.
- Platform supports multiplexed, point-of-care, and potentially wearable or fiber-based clinical diagnostic tools.

**Supplementary Information:**

The online version contains supplementary material available at 10.1007/s44258-026-00090-w.

## Introduction

Optical metasurfaces and metamaterials employ artificially engineered nanostructures to realize optical properties that are not available in nature. Leveraging their sensitivity to the surrounding dielectric environment, this novel platform can be exploited for spatiotemporal light control [1, 2] or optical biosensing applications [3]. Metasurface biosensors offer higher sensitivity and smaller form factors [4, 5], presenting immense potential from cell biology studies (monitoring membrane proteins, cell secretion [6, 7]) to clinical diagnostics and drug screening (liquid biopsies, lab-on-chip, point-of-care testings [8–11]). However, despite extensive research exploring higher performance and novel functionalities [12–14], it remains a significant challenge to translate these benchtop successes into clinical applications and commercial products [15]. One major roadblock is the heavy reliance on specialized and expensive top-down nanolithographic techniques. On the one hand, deep ultraviolet lithography is not accessible to most research laboratories, while e-beam lithography (EBL) and focused ion-beam suffer from inherently low throughput [16]. Nanoimprint lithography improves the throughput but still relies on cleanroom facilities and molds fabricated by EBL. This limitation not only prevents large-volume production, but also constrains metasurfaces to small areas compared with the typical optical interrogation spot, ultimately reducing the resonance quality factors [17] and signal-to-noise ratio from an analytical standpoint [18]. On the other hand, bottom-up techniques like nanosphere lithography (NSL) are high-throughput and low-cost, but the structural defects and poor long-range order limited the sensing performance [19, 20]. Recent advances promise high-quality or wafer-scale manufacturing, but reliable biosensing and biomedical applications have yet to be demonstrated [21–24]. In addition to scalable manufacturing, the integration of metasurfaces with microfluidic systems is essential for enabling label-free detection of low-concentration biomarkers for rapid testing and real-time monitoring. To improve the sensing performance for a lower limit of detection (LOD), it is necessary to optimize mass transport within the optofluidic integration to efficiently deliver analytes to the functionalized metasurface and maximize target capture [25–28].

Historically, plasmonic metasurfaces have been widely employed for refractometric sensing, utilizing localized surface plasmon resonance with superior surface sensitivity to the surrounding refractive index (RI), compared with conventional surface plasmon resonance [29]. Among various plasmonic sensor architectures, gold nanohole arrays (AuNHAs) [6–11] are particularly prominent due to their simple geometry and extraordinary optical transmission, in which resonant coupling to surface plasmons enables light transmission far exceeding classical predictions [30–32]. In this work, we present AuNHA metasurfaces that holistically address the challenges of high-quality, scalable fabrication, functionalization and optofluidic integration to improve the sensing performance. The resonance modes were analyzed by both theoretical calculations and numerical simulations to guide the metasurface design. A microinjection-driven NSL technique was employed to fabricate large-area, high-quality AuNHAs. We investigated how the sensing performance is affected by the geometric arrangement of the metasurface, the microfluidic channel, and the optical interrogation region. The scalable, cost-effective meta-sensors demonstrated ultrasensitive, label-free, and real-time detection of biomolecules and nanoparticles, achieving performance metrics (bulk refractive index sensitivity (BRIS), LOD, dynamic range) that rival the state-of-the-art metasurface biosensors fabricated with sophisticated top-down nanolithographic techniques.

## Methods

### Chemicals and materials

Isopropanol (IPA), sodium chloride (NaCl), cysteamine hydrochloride (98%), 3-mercaptopropionic acid (MPA, 99%), bovine serum albumin (BSA), human serum abumin (HSA), streptavidin (STP), Biotin-PEG4-NHS ester (Broadpharm), Phosphate-Buffered Saline (PBS), HEPES buffer, human immunoglobulin G (IgG), anti-human IgG (anti-IgG), Nanosphere™ size standards of diameter 600 nm (Thermo Scientific™ 3000 Series), were purchased from Fisher Scientific (Waltham, MA, USA). Ammonia solution (25%), ethyl alcohol (anhydrous, 200 proof), N-(3-Dimethylaminopropyl)-N′-Ethylcarbodiimide Hydrochloride (EDC), N-Hydroxysuccinimide (NHS), hydrogen peroxide solution (30wt% in H_2_O), trichloro-(1H,1H,2H,2H-perfluorooctyl)-silane, C-Reactive Protein were purchased from Millipore Sigma (St. Louis, MO, USA). STP coated polystyrene (PS) nanoparticle (50 nm) was purchased from Nanocs (New York, NY, USA). Photoresist SU-8 2000.5 was purchased from MicroChem (Newton, MA, USA). Polydimethylsiloxane (PDMS, Sylgard 184) and curing agent were obtained from Dow Corning (Midland, MI, USA). SU-8 2010 photoresist was purchased from Kayaku Advanced Materials (Westorough, MA, USA). Polytetrafluoroethylene (PEFE) tubing (OD 1 mm; ID 0.5 mm) was purchased from Chipshop (Jena, Germany). Deionized (DI) water was from Ultrapure Milli-Q (Merck Millipore, Burlington, MA, USA).

### Numerical simulation and data analysis

All simulations were performed by using commercial software (COMSOL Multiphysics® 6.2) to model the far-field (transmission) and near-field (electric fields) responses of the AuNHA metasurface. Under linearly polarized excitation at normal incidence, a hexagonal unit cell with periodic boundary conditions was used to simulate an infinite periodic hexagonal array in the transverse plane. Perfectly matched layer absorbing boundary conditions were applied at the top and bottom boundaries in the propagation z direction. To ensure simulation efficiency and accuracy, the minimum mesh size was set to 0.4 nm for the nanohole region.

The data collected from simulation and experiments were processed using MATLAB (MathWorks). For resonance wavelength identification, we used a centroid fitting algorithm developed by Dahlin et al. [33]. The experimental spectral data was preprocessed with a Savitzky-Golay filter to reduce noise before centroid fitting. As the optical system noise fluctuates across different measurements, we used the same optimized noise level to calculate LODs. Atomic force microscope (AFM) images were processed by Gwyddion (v2.61) and ImageJ (v1.54p).

### AuNHA fabrication and characterization

The microinjection-driven NSL technique was used by combining the micropropulsion injection with the air-glass-water interface method [34] for a high-quality, scalable and automation-compatible self-assembly process. Briefly, glass slides were cleaned in ES-7X detergent (100%) boiling in a beaker for 60 min and then rinsed with water. The PS nanospheres were first centrifuged and washed with water twice and redispersed in EtOH/H_2_O (5:5 volume ratio) to dilute to a concentration of 5 wt%. The clean slides were then submerged in a thin layer of water (~ 1.5 mL), after which the prepared PS nanospheres were slowly delivered to the edge of the slide via a syringe needle to establish a stable interface line between the mixed PS solution and water (Fig. S1a). Due to the stable injection rate (~ 3 μL/min), this process ensures a steady and controlled self-assembly until a complete monolayer is formed at the water surface. After drying, a hexagonal-close-packed (HCP) PS monolayer was formed and deposited on the glass slide. The size of the PS nanospheres was reduced and adjusted by reactive ion etching (RIE, Oxford PlasmaPro NPG80: 100 W RF power, oxygen flow 50 sccm, 60 mTorr). The etched mask was subject to e-beam evaporation, depositing 4 nm of Ti as adhesion layer and 50 nm of Au at a rate of 0.4 Å/s and 0.85 Å/s, respectively. Finally, the PS mask was removed by sonication for 1 min in Toluene solution, then 3 min each in IPA and water for removing the residues. The fabricated large-area (millimeter to centimeter scale) PS monolayers and AuNHAs were inspected in an optical microscope. The nanotopography was imaged in an AFM (DriveAFM from Nanosurf, Swiss) in dynamic mode.

### Integration with PDMS microfluidics

The microfluidic channel mold was fabricated by photolithography with a channel width of 800 µm. A 10 µm-thickness SU-8 resist (2010) film was spin-coated at 3000 rpm, followed by soft baking at 65 °C for 2 min and 95 °C for 2 min. The resist was exposed to define the channel pattern using a μMLA maskless aligner (Heidelberg Instruments) with an exposure dose of 600 mJ·cm^−2^. Post-exposure baking was performed at 65 °C for 1 min and 95 °C for 1 min. The sample was then developed in SU-8 developer for 1 min, rinsed with IPA, dried under nitrogen, and hard-baked at 150 °C for 3 min. The mold was then silanized using trichloro-(1H,1H,2H,2H-perfluorooctyl)-silane in a desiccator under vacuum for 20 min to facilitate PDMS release. PDMS prepolymer and curing agent were mixed at 10:1, cast onto the mold, and cured at 60 °C for 3 h. After demolding, inlet and outlet ports were created using a biopsy punch (1 mm inner diameter). Both the AuNHA substrate and the PDMS channel were treated with oxygen plasma (Harrick Plasma) at 40 W for 1 min to promote surface bonding. The PDMS channel was then carefully aligned and bonded to the AuNHA substrate using a stereomicroscope (Leica M80) to ensure proper positioning of the AuNHA in the channel.

### Refractometric sensing and biosensing

After the integration of PDMS microfluidic chamber and connecting PTFE tubing at the channel inlet and outlet, the optical transmission spectra were measured by a spectrometer connected to a Nikon microscope (Ti2 Inverted) with a halogen lamp. For large wavelength range scans (400 ~ 900 nm), a compact Isoplane81 spectrometer (Teledyne, CCD resolution ~ 0.52 nm) was used with 100 µm slit, 600 groves/mm grating, 20 × Objective (NA = 0.45) and an acquisition time of 1 s. For shorter wavelength range scans (700 ~ 781 nm), an HRS-500 spectrometer (Princeton Instrument, CCD resolution ~ 0.13 nm) was used to record temporal changes in transmission spectra (spectral binning at the center 10 pixels), with 25 µm slit, 600 groves/mm grating, 10 × Objective (NA = 0.3). The spectra were collected by averaging 10 frames with 1 s exposure time for each frame. The acquisition interval was 15 s for continuous recording. A background spectrum was acquired on the glass background without AuNHA. NaCl solutions with varied concentrations (NaCl (0.9%), *n* = 1.3346; NaCl (2.7%), *n* = 1.3377; NaCl (4.5%), *n* = 1.3408; NaCl (9%), *n* = 1.3487) were used to quantify the BRIS.

For surface functionalization of AuNHA with antibody, the metasurface was subjected to a slow flow (0.05 µL/min) of MPA (10 mM) aqueous solution overnight to modify the surface with a self-assembled monolayer (SAM) of bifunctional linker. After washing, a surface activation step was completed with a flow of EDC/NHS (0.1 M: 0.25 M) mixture for 45 min. Anti-IgG (100 µg/mL) was delivered to the activated AuNHA for 45 min. Following a PBS washing step, the surface was passivated with BSA (500 µg/mL) for 15 min and stored in PBS solution at 4 °C before use. For biotinylation, a simpler process started with the surface modification by a slow flow of cysteamine (10 mM) overnight. After washing, the amine-activated AuNHA was directly introduced to a slow flow of Biotin-PEG4-NHS (7.5 mM in HEPES) to react for 2 h. The metasurface was further washed with PBS, passivated with BSA (500 µg/mL) and washed again before storage in PBS at 4 °C. For the real-time sensing, IgG/STP/STP-PS concentrations from high to low were prepared by serial dilution and then injected into the microfluidic channel from low to high concentrations a at a constant flow rate of 20 µL/min, with a short PBS washing step (15 min) between consecutive concentrations. New tubing was used for different steps to avoid potential cross contamination.

## Results

### Mode analysis and rational design

**Fig. 1:**
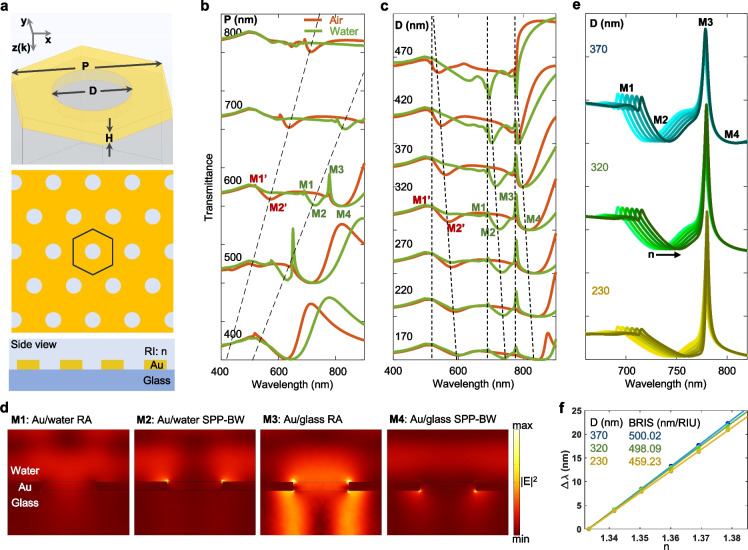
**a** Schematic diagram of gold nanohole array (AuNHA) metasurface: geometric configuration of a unit cell with plane wave incident along the z direction (k), top view and side view of the hexagonal array. P is the periodicity of the array and D is the nanohole diameter. **b** Simulated transmission spectra in both air (*n* = 1) and water (*n* = 1.3330) by sweeping P while keeping the D/P ratio at 0.5. **c** Simulated transmission spectra by sweeping D while keeping P = 600 nm. The dashed lines denote the evolution of primary resonance modes. **d** The near-field enhancement of the local electric fields in the vicinity of AuNHA unit cells (P = 600 nm, D = 320 nm) for Rayleigh’s anomaly (RA) and surface plasmon polariton-Block waves (SPP-BW) modes. **e** Simulated transmission spectral shifts when the bulk solution refractive index (RI) gradually increases from 1.3330 to 1.3786 for D = 230/320/370 nm, respectively. **f** Linear calibration curves for the simulated M2 resonance shift as a function of RI, extracted from (**e**)

In designing a high-performance and scalable metasurface, we envisioned AuNHA structures in HCP arrangement (Fig. 1a), which can be conveniently fabricated by high-throughput and cost-effective NSL. The metasurface is modeled as a hexagonal unit cell with period P, nanohole diameter D and height H. It is positioned on top of a glass substrate and covered by a dielectric layer to model biomolecular sensing in an aqueous environment. The 0th order transmission spectra in both air and water were systematically studied by simulation. First, the period P was swept from 400 to 800 nm, while keeping the ratio of D/P at ~ 0.5 (Fig. 1b). The major transmittance dips (M2’ mode in air, M2 mode in water) showed a redshift with increasing P. P = 600 nm was chosen for a relative high amplitude of M2 mode in the visible spectrum. Then, the nanohole diameter D was swept from 170 to 470 nm, showing a series of dips (M2’, M2, M4) and peaks (M1’, M1, M3) (Fig. 1c).

Theoretically, nanohole arrays support two types of anomalies: an “edge” anomaly at the passing of a diffraction order, i.e., Rayleigh’s anomaly (RA), which appears as sharp peaks; and a “diffuse” anomaly associated with the excitation of the surface plasmon polariton-Block waves (SPP-BW), which appears as relatively broad dips [35–37]. These modes should strictly satisfy the momentum matching condition (SPP-Bragg’s equation) between the in-plane wavevectors of the incident light and that of the reciprocal lattice vectors (e.g., a hexagonal array in this case):

1 $$\left|{k}_{0}{n}_{inc}sin\theta +{G}_{i,j}\right|= {k}_{SPP}\ or\ {k}_{RA}$$

for the SPP-BW and RA modes respectively, where ${k}_{0}{,n}_{inc}, \theta$ are the free space wavevector, RI of the dielectric layer, and incident angle, ${G}_{i,j}$ is the grating momentum for the scattering order (i,j). At normal incident, this equation can be simplified to the following [36, 37]:

2 $${\lambda}_{SPP}\left(i,j\right)= \frac{\sqrt{3}}{2}P\frac{\sqrt{{\varepsilon}_{Au}{\varepsilon}_{d}}}{\sqrt{{\varepsilon}_{Au}{+\varepsilon }_{d}}}\frac{1}{\sqrt{{i}^{2}+ij+{j}^{2}}}$$

3 $${\lambda}_{RA}\left(i,j\right)= \frac{\sqrt{3}}{2}P\sqrt{{\varepsilon}_{d}}\frac{1}{\sqrt{{i}^{2}+ij+{j}^{2}}}$$

where ${\varepsilon}_{Au}$ is the permittivity of Au described by the Drude model, ${\varepsilon}_{d}$ is the permittivity of dielectric, either the glass substrate or medium, e.g., air, water. Following the equations, theoretical resonance wavelength positions are predicted (λ_theo_ for P = 600 nm) to precisely assign the modes to simulated spectra (λ_sim_ for P = 600 nm, D = 320 nm), as labeled in Fig. 1c and compared in Table 1.

**Table 1:** Resonance modes and wavelengths by analytical calculation and numerical simulation

Mode	M1’ Au/air ${\lambda}_{RA}\left(\mathrm{1,0}\right)$	M2’ Au/air ${\lambda}_{SPP}(\mathrm{1,0})$	M1 Au/water ${\lambda}_{RA}\left(\mathrm{1,0}\right)$	M2 Au/water ${\lambda}_{SPP}(\mathrm{1,0})$	M3 Au/glass ${\lambda}_{RA}\left(\mathrm{1,0}\right)$	M4 Au/glass ${\lambda}_{SPP}\left(\mathrm{1,0}\right)$
λ_theo_ (nm)	519	563	693	727	779	814
λ_sim_ (nm)	519	573	690	729	776	818

The resonance peaks M1’, M1 and M3 maintained constant wavelength positions despite changes in D (Fig. 1c), as expected for RA modes that do not depend on the metal dispersion function according to Eq. (3). In contrast, there is a universal blueshift in M2’, M2 and M4 modes with increasing D, as the SPP index term ($\frac{\sqrt{{\varepsilon}_{Au}{\varepsilon}_{d}}}{\sqrt{{\varepsilon}_{Au}{+\varepsilon }_{d}}}$) in Eq. (2) is gradually reduced with a higher nanohole area fraction (i.e., the AuNHA surface becomes highly perforated and behaves more like a lower-RI material than a continuous Au film). In water, it is evident that RA peaks M1 and M2 are always sharper than SPP-BW dips M2 and M4. As D increases, the spectral widths of M2 and M4 decrease, indicating stronger hybridization of these SPP-BW modes with either the RA continua or the nanohole cavity modes. Moreover, Fig. 1d shows the near-field intensity of the normalized electric field intensities for mode M1, M2, M3 and M4 in the vicinity of a nanohole within the x–z cross-section of a hexagonal unit cell (P = 600 nm, D = 320 nm). The field intensity distributions of these modes are consistent with their character assignments: for M1 and M3 (RA modes), they feature a relatively extended distribution over the top (Au/water) and bottom (Au/glass) interfaces respectively. For M2 and M4 (SPP-BW modes), they show a more localized field distribution at the edges of the holes as it is coupled to the nanohole dipolar excitation at the Au/water and Au/glass interfaces respectively. The RA-dominated M3 mode also exhibits contributions from SPP-BW as evidenced by the enhanced near-fields at the Au/water edges.

For the modeling of refractometric sensing, we further simulated the transmission spectra as the bulk solution RI was scanned from 1.3330 to 1.3786 (Fig. 1e). The Au/water modes M1 and M2 show clear redshifts with increasing RI, whereas Au/glass modes M3 and M4 maintained constant wavelengths. The BRIS, calculated from the centroid shifts of M2 resonance, increases with D and eventually reaches a saturated level (Fig. 1f): the BRIS 498 nm/refractive index unit (RIU) at D = 320 nm is quite close to 500 nm/RIU at D = 370 nm. Further increasing D toward P may cause cracks and discontinuities in the AuNHA film, leading to deviations from the desired resonances [38]. Therefore, the geometric parameters (P = 600 nm, D = 320 nm) were selected as the optimal design. Meanwhile, the theoretical wavelength positions, simulated spectra characteristics, field distributions, and spectral shifting behaviors with respect to geometric parameters and medium RI, are all in good agreement, collectively validating the mode assignments (see detailed discussion in Supplementary Note).

### Device fabrication and spectral characterization

**Fig. 2:**
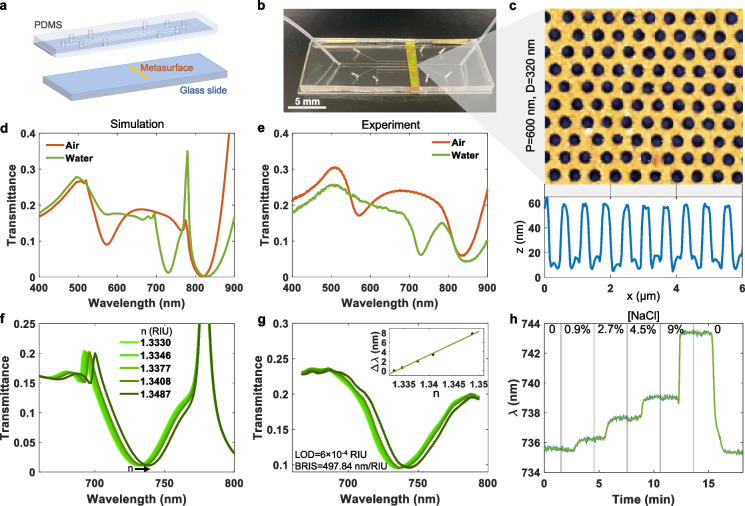
**a** Schematic of the microfluidic integration on AuNHA metasurface. **b** Optical photograph of the integrated optofluidic meta-sensor. **c** Zoom-in of the AuNHA metasurface: atomic force microscopy image with a representative line profile shown at the same scale. **d** Simulated transmission spectra and **e** Experimentally measured spectra in both air and water. **f** Simulated spectral shifts and **g** Experimental measured spectral shifts as the solution RI gradually increases from 1.3330 to 1.3487. **h** Real-time sensorgram recording the centroid wavelength as the overflowing NaCl solution concentration changes stepwise

The AuNHA metasurfaces were fabricated using a high-throughput NSL process (Fig. S1a). Through a water–air-glass interface method [34], uniform HCP PS nanosphere monolayer was formed. The monolayer was then subject to O_2_ RIE to reduce nanospheres to the desired size and create masks for metal deposition. After depositing 4 nm of Ti and 50 nm of Au thin film, the nanosphere masks were removed to obtain AuNHAs. The fabricated metasurface was integrated with a PDMS microfluidic chamber as a flow cell for sensing (Fig. 2a, b; Fig. S2). The high-quality long-range order of the AuNHA is validated by both AFM imaging (Fig. 2c) and optical photographs (Fig. S1b). The nanohole diameter D was adjusted by the RIE time (D = 370/320/230 nm, see the AFM images, line profiles, and histograms of the respective D values in Fig. S3). Across all nanohole diameters, the histograms demonstrate good uniformity, along with a distribution arising from the 5–10% size variation of the PS nanospheres.

For the microfluidic-integrated metasurface with optimized parameters (P = 600 nm, D = 320 nm), the transmission spectra were measured in both air and water environments, and comparison with simulations (Fig. 2d, e). In AuNHA metasurfaces, long-range order provides higher transmission efficiency, quality factor, and signal-to-noise ratio than short-range order [39, 40], which likely underlies the close correspondence between experiment and simulation achieved with our improved nanopatterning quality. Moreover, the spectra measured among different samples were highly consistent (Fig. S4), further confirming the high uniformity, precision, and repeatability of the scalable fabrication. Next, refractometric sensing was demonstrated by injecting aqueous solutions ranging from DI water to varying NaCl concentrations. The experimental measured M2 resonance and its spectral shift closely matched the simulations, whereas the M1, M3 narrow peaks were not fully resolved with our cost-effective optical setup using a halogen lamp (Fig. 2f, g). This experimentally justifies our selection of the M2 Au/water (1,0) SPP-BW resonance as the major mode for refractometric sensing. A linear curve fit of the M2 dip centroid at corresponding RI (inset in Fig. 2g) shows an experimental BRIS of 497.84 nm/RIU, almost identical to the simulation result (Fig. 1f). A temporal record of spectral centroid position captured the real-time evolution of bulk RI, as it gradually increased with NaCl concentration and then returned to DI water (Fig. 2h). The recovered spectral position of DI water confirms the system stability with negligible signal drift, which is essential for ultrasensitive detection of low-concentration analytes, and for real-time, continuous monitoring. Again, this stability is attributable to the high patterning uniformity, which makes the collected spectra robust against mechanical or thermal drift during continuous acquisition. Overall, the experimental results in this section closely match the simulated spectra and spectral-shift behaviors, further corroborating our mode analysis and rational design presented in the previous section.

### Biomolecular sensing demonstrated by IgG

**Fig. 3:**
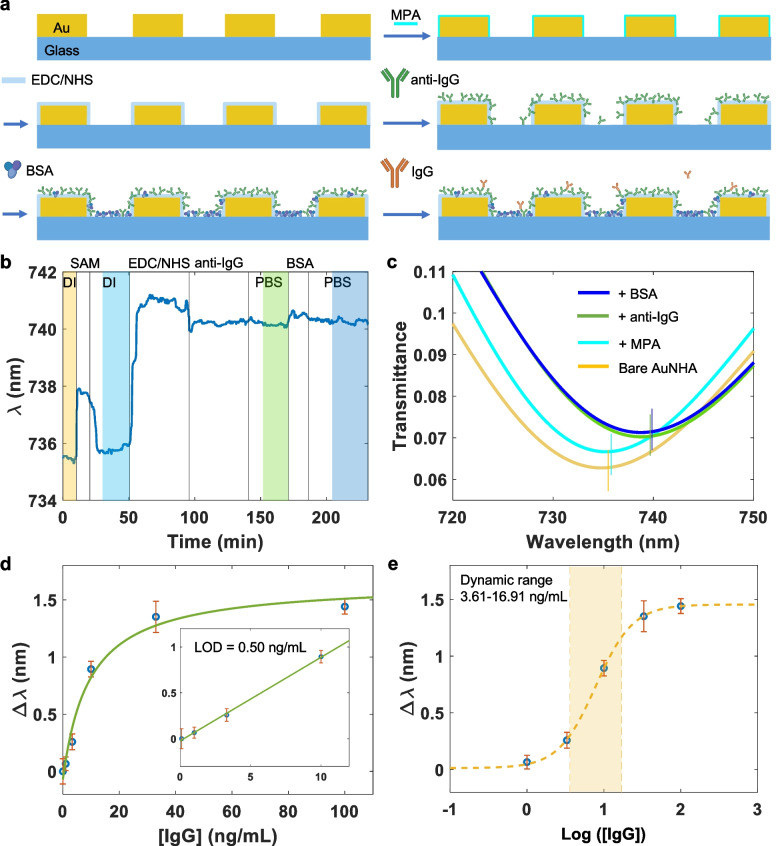
**a** Schematic of the step-by-step functionalization, passivation and biosensing of immunoglobulin G (IgG): A self-assembled monolayer (SAM) of 3-mercaptopropionic acid (MPA) is formed on the AuNHA metasurface, followed by Ethylcarbodiimide Hydrochloride (EDC) and N-Hydroxysuccinimide (NHS) activation to immobilize anti-IgG for subsequent capture of the target IgG. The metasurface is passivated by bovine serum albumin (BSA) to prevent nonspecific adsorption. **b** Real-time sensorgram recording the centroid wavelength during the functionalization and passivation process. **c** Averaged spectra after each functionalization step corresponding to the colored regions in (**b**). The centroid shift vs. IgG concentration: **d** Langmuir fit and linear fit (inset) for the low-concentration range to evaluate the limit of detection (LOD); **e** S curve fitted by a 4-parameter logistic model. Each data point in (**d**) and (**e**) represents the average over a stable Phosphate-Buffered Saline (PBS) elution period after each IgG injection, with error bars indicating the standard deviation (*n* = 41 consecutive measurements)

For biosensing, the proof of concept was demonstrated by the detection of human IgG, one of the most-commonly screened immune-serological targets for its significance in indicating disease immunity, past infection, and autoimmune conditions. First, a multi-step metasurface functionalization was implemented by flowing respective reagents through the microfluidic channel while monitoring the centroid shifts in real time (Fig. 3a). A sensorgram was thus recorded to track and confirm the successful covalent binding of each linker or capturing ligand to the metasurface (Fig. 3b). In a zoomed-in view of the averaged spectra (Fig. 3c), there were ~ 0.5 nm red-shift after the MPA SAM conjugation, ~ 3.5 nm redshift following the binding of the capturing agent anti-IgG via EDC/NHS, and only ~ 0.3 nm for BSA passivation. The shifts induced by surface functionalization are consistent with those reported in previous work on a nano-plasmonic sensor [8]. The relatively small shift after BSA passivation indicates good anti-IgG coverage, leaving little available surface area for nonspecific BSA adsorption. Furthermore, IgG solutions with gradually increasing concentrations were injected onto the functionalized metasurface, with PBS elution performed after each concentration, resulting in a resonance redshift that followed a Langmuir isotherm curve (Fig. 3d). In the low-concentration regime of 0 ~ 10 ng/mL, a linear calibration curve can be fitted to the centroid shifts (Fig. 3d inset). The LOD for IgG was calculated to be LOC $=3\upsigma /k=$ 0.5 ng/mL, where k is the slope of the linear fit, the standard deviation $\sigma$ was calculated from the centroid variations while flowing blank PBS solution through the metasurface for 15 min. The full data till saturation were fitted with a 4-parameter logistic model that is characterized by the typical S-shaped sigmoid for ligand-receptor binding and dose response curves (Fig. 3e), with a dynamic range of ~ 0.67log, which is also comparable to state-of-the-art devices fabricated by top-down lithography [41, 42].

###  Optimization of functionalization and microfluidics

**Fig. 4:**
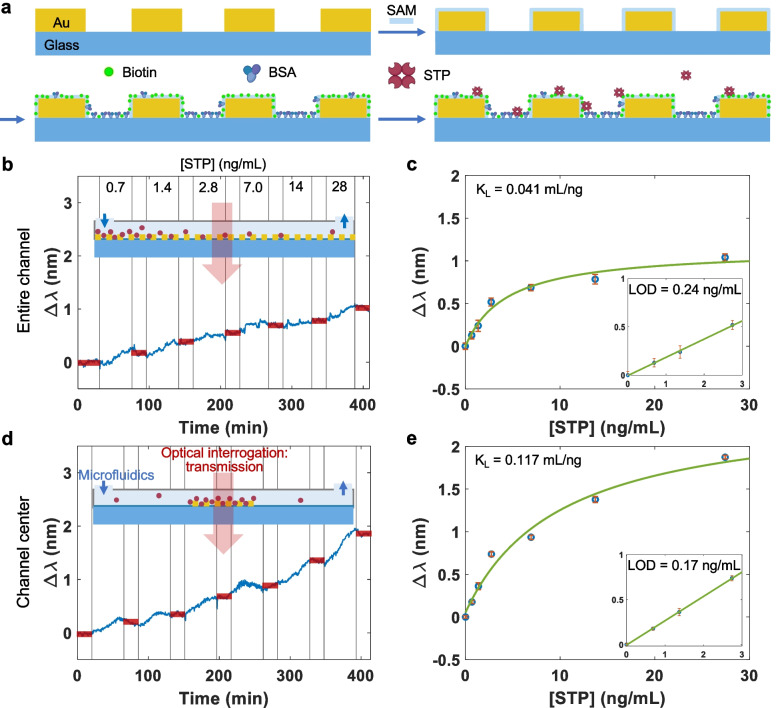
**a** Schematic of the step-by-step functionalization, passivation and biosensing process using streptavidin (STP)-biotin interactions as a model: A SAM of cysteamine is formed on the AuNHA metasurface, to immobilize NHS-biotin for subsequent capture of the target STP, with BSA passivation. Entire channel configuration: **b** Real-time sensorgram recording the centroid wavelength shift during alternating micro-injections of STP at gradually increasing concentrations, followed by PBS elution (highlighted in red). **c** Langmuir fit and linear fit (inset for the low-concentration range with LOD evaluation) of the centroid shift vs. STP concentration from (**b**). Channel center configuration: **d** Real-time sensorgram recording the centroid wavelength shift during alternating micro-injections of STP at gradually increasing concentrations, followed by PBS elution (highlighted in red). **e** Langmuir fit and linear fit (inset for the low-concentration range with LOD evaluation) of the centroid shift vs. STP concentration from (**d**). Each data point in (**c**) and (**e**) represents the average over a stable PBS elution period, with error bars indicating the standard deviation (*n* = 21 consecutive measurements)

Taking a closer look, the sensing performance of microfluidic-integrated sensors are influenced by functionalization efficiency and mass transport process within the microfluidic system [25–28]. To further improve the functionalization efficiency, a cysteamine SAM (Fig. 4a) can potentially yield higher surface coverage than the MPA SAM which requires an additional EDC/NHS step (Fig. 3a). A microfluidic channel with a small height of 10 µm was integrated to facilitate analyte delivery to the metasurface (Fig. S2). More importantly, the binding reaction, governed by diffusion and convection, can produce an adsorption gradient along the flow direction [43]. The analyte depletion can reduce the effective adsorption rate (${k}_{on}$), and consequently the Langmuir adsorption constant (${\mathrm{K}}_{L}=\frac{{k}_{on}}{{k}_{off}}$). The desorption rate (${k}_{off}$) and maximum adsorption capacity (Q_max_) stay unaffected, as they are intrinsic properties determined by the molecules and the surface. As a result, the sensing performance of a metasurface that extends across the entire channel is limited, because analyte capture is highest at the channel inlet, whereas the optical interrogation spot is typically located at the center of the channel. The adsorption constant and sensing performance can be improved by confining the analyte capture to the interrogation region, which maximizes the detected signal. To achieve this, the metasurface should be either patterned or selectively functionalized within a region slightly larger than the interrogation spot, while the remainder of the channel is passivated to prevent nonspecific adsorption [41, 44].

To test this hypothesis and demonstrate the versatility of our platform, we used high-affinity biotin-STP binding as a model system. The metasurface was functionalized by a SAM of cysteamine to immobilize NHS-biotin for subsequent capture of the target STP (Fig. 4a). Two distinct metasurface configurations were implemented. (1) Entire channel: the metasurface extended across the entire channel (Fig. 4b, c); (2) Channel center: the metasurface was patterned as a 1 mm-wide stripe oriented orthogonally through the center of the channel, while the rest of channel was passivated by BSA (Fig. 4d, e). Similarly to IgG sensing, STP solutions with gradually increasing concentrations were injected. The centroid shifts were monitored in real time and recorded as sensograms (Fig. 4b, d). The spectral redshifts were averaged for each concentration, yielding Langmuir isotherm curves (Fig. 4c, e). Extracted from the Langmuir isotherm fits (${\mathrm{q}}_{e}=\frac{{Q}_{\mathrm{m}\mathrm{a}\mathrm{x}}* {K}_{L}*[STP]}{{1+ K}_{L}*[STP]}$) over a broader concentration range, the ${\mathrm{K}}_{L}$ was ~ 3 times higher in the “channel center” configuration than the “entire channel”, suggesting more efficient capture of STP molecules validating our hypothesis. The “channel center” configuration provided higher signals and a lower LOD than the “entire channel”, based on the linear fits in the low concentration range (insets in Fig. 4c, e). In both configurations, the LODs for STP were lower than that for IgG, likely because biotin-STP binding has a stronger affinity than the interaction between anti-IgG and IgG [9]. Meanwhile, by reducing functionalization steps, the optimized cysteamine-based scheme is also potentially more efficient than the previous MPA-based scheme.

### Biosensing of nanoparticles

**Fig. 5:**
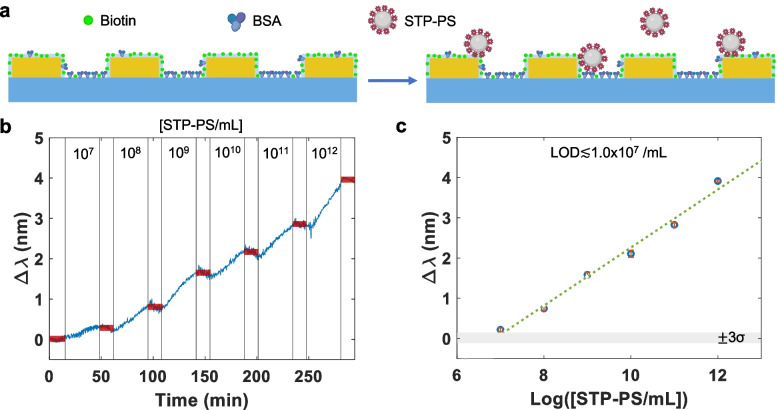
**a** Schematic of the functionalization, passivation and biosensing process using polystyrene (PS) nanoparticles as a model: A SAM of cysteamine is formed on the AuNHA metasurface, to immobilize NHS-biotin for subsequent capture of the target STP-PS nanoparticles, with BSA passivation. **b** Real-time sensorgram recording the centroid wavelength shift during alternating micro-injections of STP-PS nanoparticles at gradually increasing concentrations, followed by PBS elution (highlighted in red). **c** Linear fit of the centroid shift vs. STP-PS nanoparticle concentration from (**b**) on a logarithmic scale. Each data point represents the average over a stable PBS elution period, with error bars indicating the standard deviation (*n* = 21 consecutive measurements); 3σ represents the noise level of blank PBS solution. The signal at the lowest concentration of 10^7^/mL is above the 3σ level

In addition to different biomolecules, our metasurface biosensor can be customized to detect various targets of virus particles and extracellular vesicles (EVs). Again, the AuNHA metasurface was functionalized with biotins, to detect STP coated PS nanoparticles (STP-PS, D = 50 nm), which serve as a common model system to mimic virus particles (20–300 nm) [45, 46] and EVs (30–150 nm) [8] in the similar size range (Fig. 5a). The STP-PS solutions were injected to an integrated sensor from low to high concentrations. Each microinjection of STP-PS was followed by a PBS elution to remove loosely bound or non-specifically adsorbed particles. The entire sequence was monitored by continuously tracking the centroid shifts. A calibration curve can be established by extracting the average centroid shifts against the logarithms of corresponding nanoparticle concentrations, which shows a linear fit (Fig. 5c). The gray shade indicates $\pm 3\sigma$ noise level for $\Delta \lambda$. The lowest STP-PS nanoparticle concentration (10^7^/mL or 16.6 fM) injection onto the AuNHA sensor led to a signal change that is above the $3\sigma$ level. Therefore, we estimate that the LOD for the detection of STP-PS is below 10^7^/mL with the AuNHA metasurface. The high sensitivity showcased here, together with a large dynamic range (i.e., 5 orders of magnitude of nanoparticle concentrations). Compared with the STP-PS model, detecting biological particles is more challenging because they generally exhibit lower refractive indices and greater surface heterogeneity, with variable distributions of proteins, lipids, glycans, and other biomolecules, in addition to the target biomarkers. Nevertheless, this work demonstrates clinical relevance for EV detection, as cancer related EV concentrations range from 10^9^ and 10^10^/mL depending on tumor burden [47].

## Discussion

In summary, we have demonstrated scalable and versatile AuNHA metasurface biosensors capable of detecting a broad range of targets from biomolecules of IgG, STP, to STP-PS nanoparticles. Through comprehensive mode analysis by both theoretical calculations and numerical simulations, we identified the Au/water (1,0) SPP-BW resonance as the most sensitive mode and optimized the metasurface geometric parameters. Compared with earlier work using NSL [19], we achieved uniform, high-quality nanopatterning over millimeter to centimeter scale (Figs. S1b, S4), leading to improved signal-to-noise ratio, repeatability and stability. We also optimized the functionalization scheme for higher efficiency and surface coverage, and investigated the influence of metasurface placement within the microfluidic channel on mass transport and sensing performance. Patterning or selectively functionalizing the metasurface only within the optical interrogation region (e.g., at the center of the channel) significantly improved the adsorption constant compared with metasurfaces extending across the entire channel.

By combining improved optical design, scalable nanopatterning, functionalization schemes and optofluidic integration, the meta-sensor achieves BRIS (498 nm/RIU at 736 nm wavelength) and LOD (0.17 ng/mL for biomolecules, ≲1 × 10^7^/mL for nanoparticles). Due to a lack of standardization of the target analytes, detection formats, optical configurations, data collection and quantification methods, comparisons of sensor LOD values provide only a rough indication of the system performance rather than definitive superiority. Here, to the best of our knowledge, we selected representative studies on AuNHA metasurfaces that used similar target biomolecules (mostly IgG) and EVs (including EV-mimicking nanoparticles), incoherent halogen lamp illumination with spectrometer-based detection, and LOD quantification based on the $3\sigma$ noise level (Table 2). At substantially reduced cost (estimated to be three orders of magnitude lower for NSL than EBL, Table S1), our scalable metasurfaces achieved LODs comparable to, or even surpass, state-of-the-art devices fabricated using top-down lithography. Moreover, operation in the visible spectrum is more convenient than in the near-infrared (NIR). The precision and stability enable real-time and continuous monitoring, which is more powerful than discrete endpoint measurements in air [48, 49] or solution [50]. Overall, the scalable manufacturing and cost-effective optical interrogation—without reliance on cleanroom facilities, coherent light or infrared optics—are promising for bridging current gaps in large-volume production and broader adoption.

**Table 2:** Comparison of AuNHA meta-sensors

Reference, year	Fabrication	λ(nm); BRIS (nm/RIU)	Surrounding medium	Target	LOD
Ref [48], 2019	Electron beam lithography	870 (NIR); 573	Air	IgG	0.2 ng/mL
Ref [49], 2022	Nanoimprint lithography	694 (VIS); 430	Air	IgG	70 ng/mL
Ref [50], 2015	Electron beam lithography	899.5 (NIR); 671	PBS endpoint	IgG	0.7 ng/mL
Ref [51], 2017	Deep ultraviolet lithography	860 (NIR); NA	PBS real-time	VEGF	0.15 ng/mL
Ref [19], 2012	Nanosphere lithography	710 (VIS); 530	PBS real-time	IgG	15 µg/mL
Ref [8], 2014	Focused ion beam lithography	610 (VIS); NA	PBS endpoint	EV	670 aM
Ref [52], 2022	Electron beam lithography	624 (VIS); 458	PBS endpoint	STP-PS (70 nm)	1.0 × 10^7^/mL
This work	Nanosphere lithography	736 (VIS); 498	PBS real-time	IgG	0.5 ng/mL
				STP	0.17 ng/mL
				STP-PS (50 nm)	≲1.0 × 10^7^/mL

Although demonstrated by plasmonic metasurfaces of AuNHA, our methods can be extended to dielectric metasurfaces, e.g., Si nanohole array [53], and other types of nanopatterns. In the future, the microfluidic configuration can be further optimized to maximize target capture [25–28] and to enable multiplexed sensing of different targets within the same sample [8]. Combining this meta-sensor with our recently developed membrane transfer and integration techniques [54, 55] will enable prospective applications from flow-through optofluidic meta-sensors to lab-on-fiber technology. Spectrometer-free, intensity-based sensing will facilitate system miniaturization for portable devices using light-emitting diode (LED) illumination [24]. Real-time, continuous monitoring could be extended from existing cell biology studies [6, 7] to wearable devices, through the integration with organic light-emitting diode (OLED) sources and organic photodiode (OPD) detectors [56], and regeneration strategies that modulate the interactions between binding ligands and target analytes [57]. The regeneration efficiency, storage stability, and long-term signal drift should be addressed for future practical deployment. Finally, the scalable nano-biopatterning technique developed in this work can also be applied for broader biomedical fields beyond biosensing, ranging from mechanobiology to mechanomedicine [58–60].

## Supplementary Information

Supplementary Material 1.

## Data Availability

The data that support the findings of this study are available from the corresponding author upon reasonable request.
