# Supplementary material for: Scalable optical metasurfaces for ultrasensitive, label-free and real-time biosensing
Source: Med X. 2026 Jul 14;4(1):19. doi: 10.1007/s44258-026-00090-w (PMC13368885; doi:10.1007/s44258-026-00090-w)
Supplement: Supplementary file 1 — Supplementary Material 1. [file 44258_2026_90_MOESM1_ESM.pdf]

Supplementary information

Scalable optical metasurfaces for ultrasensitive, label-free and real-time biosensing

Hao Wang[1,2], Nanzhong Deng[1,3], Yue Xiao[1,3], Ashish Pandey[1,2], Shunzhi Wang[4], Haogang Cai[1,2,3,*]

1 – Tech4Health Institute, New York University Grossman School of Medicine, Queens, NY, USA

2 – Department of Radiology, New York University Grossman School of Medicine, New York, NY, USA

3 – Department of Biomedical Engineering, New York University, Brooklyn, NY, USA

4 – Institute for Systems Genetics, New York University Grossman School of Medicine, New York, NY, USA

*Email: haogang.cai@nyu.edu

Supplementary Note: Mode assignments for the SPP-BW and RA modes.

By comprehensive analysis via analytical calculation, numerical simulation, and experimental observation, we have attributed the M2 mode (resonant λ ~ 730nm) to Au/water (1,0) SPP-BW mode, which is used for biosensing purposes. This mode assignment is based on the following supporting evidence.

1) Theoretical calculations help assign these modes to the simulated spectra with precisely matched resonance wavelengths as shown in Table 1.

Theoretically, nanohole arrays support two types of anomalies. These modes strictly satisfy the momentum matching condition (SPP-Bragg's equation) between the in-plane wavevectors of the incident light and that of the reciprocal lattice vectors (e.g., a hexagonal array in this case). Following the equations, theoretical resonance wavelength positions are predicted analytically (λ_{theo} for P=600 nm) to precisely assign the modes to the COMSOL simulated spectra (λ_{sim} for P=600 nm, D=320 nm), as labeled in Fig. 1c and compared in Table 1.

2) The simulated spectra match the theoretical mode characteristics.

According to references [35-37], RA modes are sharp peaks and SPP-BW modes are broad dips: an "edge" anomaly at the passing of a diffraction order, i.e., Rayleigh's anomaly (RA), which appears as sharp peaks; and a "diffuse" anomaly associated with the excitation of the surface plasmon polariton-Block waves (SPP-BW), which appears as relatively broad dips. The simulated spectra feature such characteristics. In water, it is evident that RA peaks M1 and M3 are always sharper than SPP-BW dips M2 and M4."

3) Simulated field distributions demonstrate the enhanced field intensities near the interfaces in agreement with the mode assignments.

The field intensity distributions of these modes are consistent with their character assignments: for M1 and M3 (RA modes), they feature a relatively extended distribution over the top (Au/water) and bottom (Au/glass) interfaces respectively. For M2 and M4 (SPP-BW modes), they show a more localized field distribution at the edges of the holes as it is coupled to the nanohole dipolar excitation at the Au/water and Au/glass interfaces respectively.

4) The behavior of spectral shifting to geometric parameters matches the theoretical equations.

The resonance peaks M1', M1 and M3 maintained constant wavelength positions despite changes in D (Fig. 1c), as expected for RA modes that do not depend on the metal dispersion function according to equation (3). In contrast, there is a universal blueshift in M2', M2 and M4 modes with increasing D, as the SPP index term ($\frac{\sqrt{\varepsilon_{Au}\varepsilon_d}}{\sqrt{\varepsilon_{Au}+\varepsilon_d}}$) in equation (2) is gradually reduced with a higher nanohole area fraction (i.e., the AuNHA surface becomes highly perforated and behaves more like lower-RI material than a continuous Au film).

5) The behavior of spectral shifting to surrounding refractive index matches the mode interfaces.

The Au/water modes M1 and M2 show clear redshift with increasing RI, whereas Au/glass modes M3 and M4 maintained constant wavelengths, as shown in Fig. 1e.

6) Experimental results match the simulated spectra and spectral shifting behaviors.

The experimentally measured M2 resonance and its spectral shift closely matched the simulations, whereas the M1, M3 narrow peaks were not fully resolved with our cost-effective optical setup

using a halogen lamp (Fig. 2f-g). This experimentally justifies our selection of the M2 Au/water (1,0) SPP-BW resonance as the major mode for refractometric sensing.

7) Our conclusion is corroborated by experimental validation in literature.

An angle dependent measurement with p-polarized light, but not s-polarized light, could provide further evidence for the identification of the SPP-BW mode, which has been demonstrated by an earlier work (Reilly et al. Controlling the Optical Properties of Plasmonic Disordered Nanohole Silver Films. ACS Nano, 2010, 4, 2, 615), which agrees with our results.

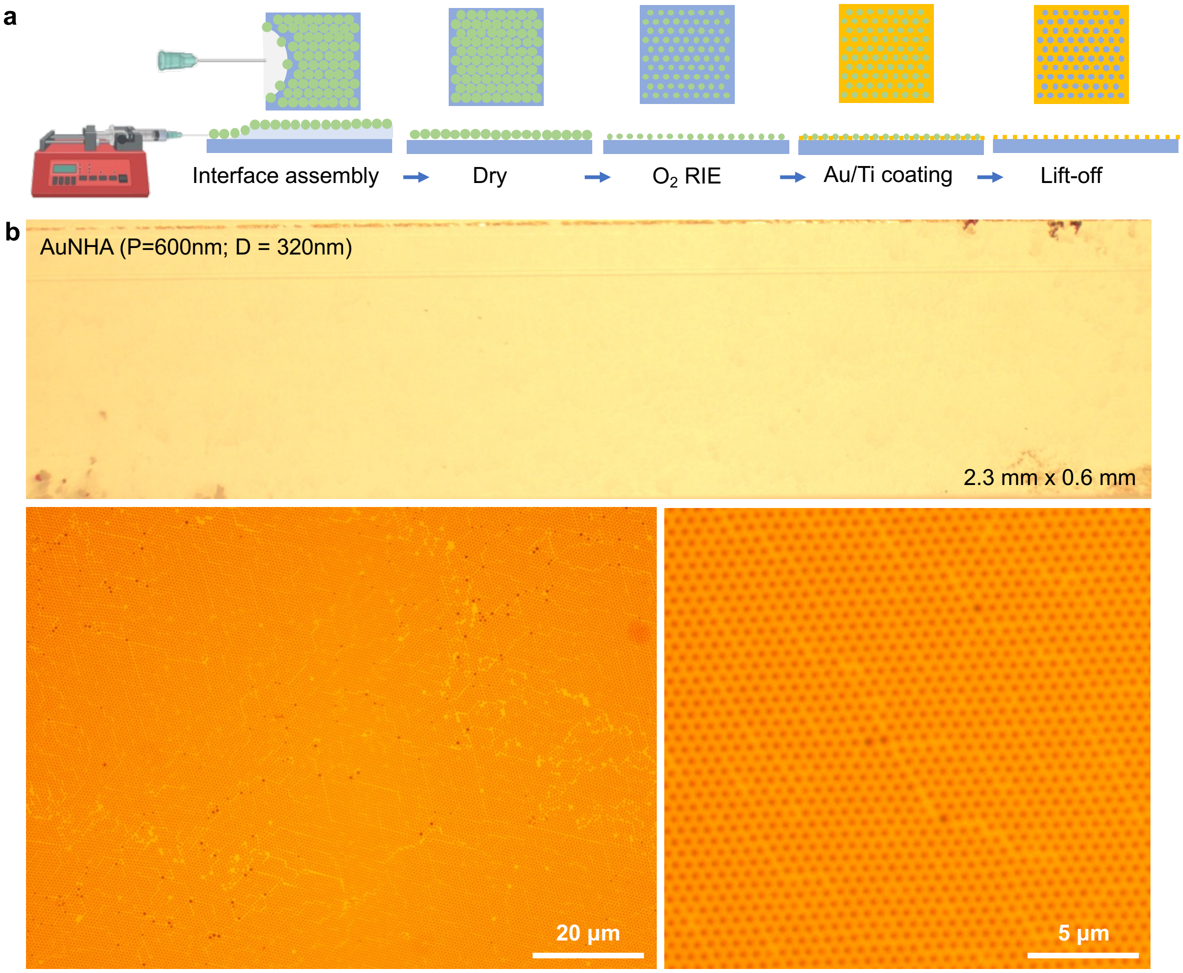

Figure S1. (a) Schematic of the micropropulsion injection driven NSL technique at the water-air-glass interface for a highly scalable fabrication of high-quality close-packed hexagonal monolayer of polystyrene nanospheres and follow-up processes to convert the NSL pattern to AuNHA metasurfaces. (b) Representative optical photographs of the fabricated AuNHA metasurface (P=600nm; D=320nm) with sufficient long-range order for biosensing applications. The zoom-out image shows a metasurface area of 2.3 mm x 0.6 mm, demonstrating large-area, high-quality patterning, e.g., in the millimeter to centimeter scale.

Glass slide:
25 mm x 75 mm

AuNHA stripe
1 mm x 14mm

Cross-
section

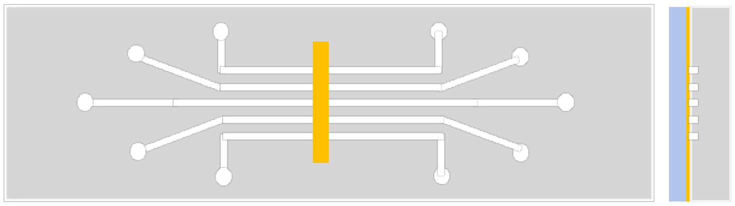

Channel parameters
h=10 μm, w=800 μm, L=50 mm
Channel volume: 0.4 μL
Gap width between channels: 2mm
PDMS layer thickness: 5mm

Figure S2. PDMS microfluidic chamber design. The channel height is limited to 10 μm to ensure efficient mass transport to the metasurface, as well as chamber integrity by avoiding high pressure drops within the channel.

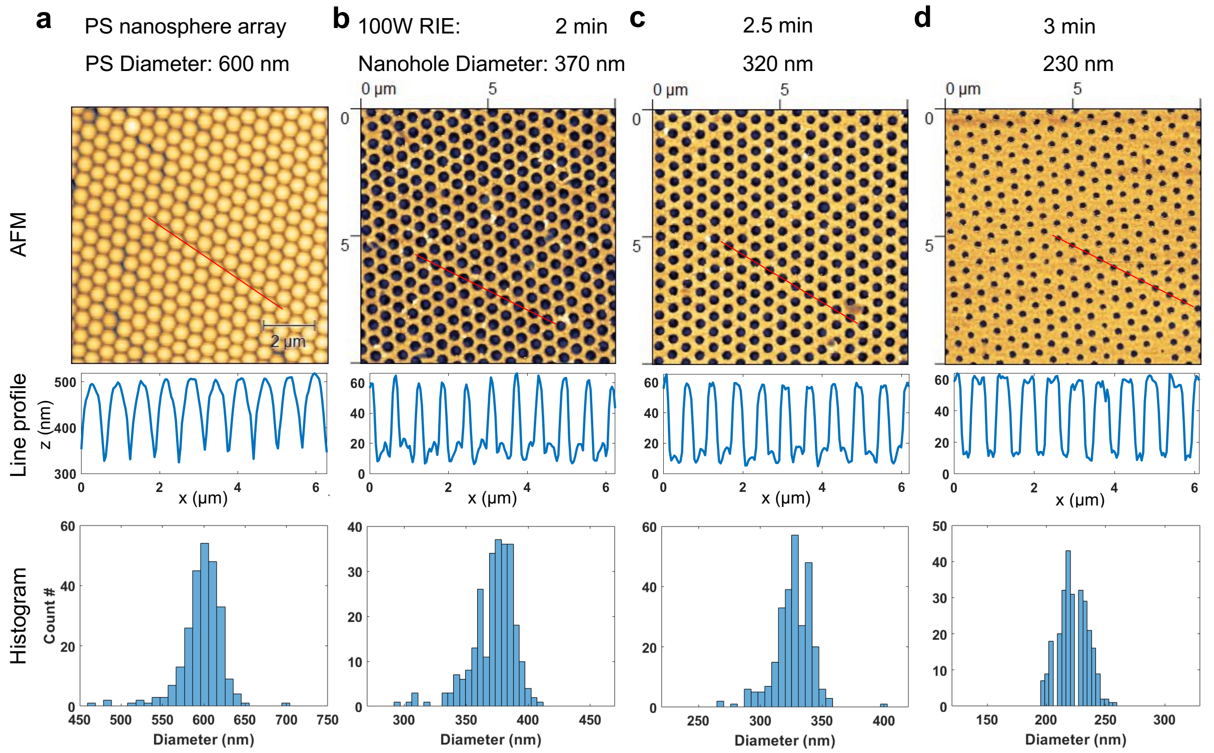

Figure S3. (a) Representative AFM image of the fabricated NSL mask, a linecut was used to show the periodicity matches that of the nanosphere diameter (D=600nm). AuNHA metasurfaces with various hole diameter D adjusted by the RIE time: (b) D=370nm after 2 min RIE, (c) D=320nm after 2.5 min RIE, (d) D=230nm after 3 min RIE. Using AFM images, the nanohole diameter D was measured by both line-profile analysis and image processing (ImageJ), with the statistical distribution summarized in histograms.

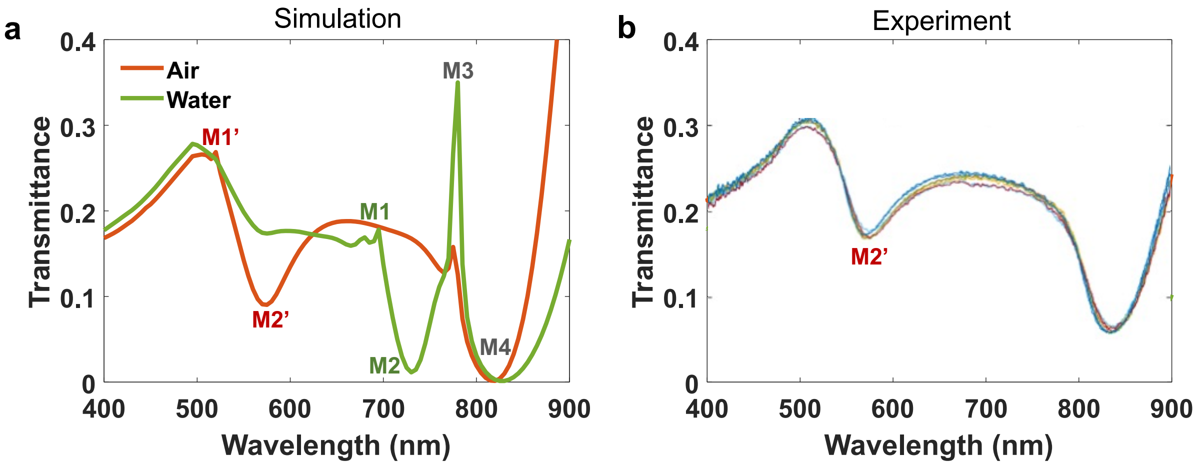

Figure S4. (a) Simulated transmission spectra of AuNHA metasurface (P=600 nm, D=320 nm) both in air and in water. The Au/water modes (M1’ and M2’ in air, M1 and M2 in water) show clear redshift with increasing RI, whereas Au/glass modes M3 and M4 maintained constant wavelengths. (b) Comparison of spectra measured among different samples, which are in agreement with the simulation and highly consistent with each other. For the sensing mode M2’ in air, the coefficient of variance is 0.9% for the centroid wavelength, and 3.0% for the dip intensity.

Table S1. Cost estimates for metasurface manufacturing: EBL vs. NSL

Cost	EBL	NSL
Facility	\$70/hr x 12hr = \$840	0
Material	PMMA: \$435/100 = \$4<br>Developer: \$106/25 = \$4	PS beads: \$725/4500 = \$0.16
Time	12 hr	1 hr
Total cost / cm^2	\$848	\$0.16

Table S1 provides a rough estimate of the metasurface manufacturing cost per cm^2, focusing on the minimum nanopatterning for EBL and NSL. The cost of the remaining processes and materials, including RIE (Au etching or PS nanosphere etching), Au deposition processes are comparable for both approaches. Overall, the nanopatterning cost of EBL is approximately 5,000 times higher than that of NSL. Our NSL-based approach is faster, more cost-effective, and therefore more scalable for high-volume production.
